# The Role of MicroRNAs in HIV Infection

**DOI:** 10.3390/genes15050574

**Published:** 2024-04-29

**Authors:** Nicolas Morando, Mara Cecilia Rosenzvit, Maria A. Pando, Jens Allmer

**Affiliations:** 1 Instituto de Investigaciones Biomédicas en Retrovirus y Sida (INBIRS), Consejo Nacional de Investigaciones Científicas y Técnicas (CONICET)-Universidad de Buenos Aires, Buenos Aires 1121, Argentina; nicolasmorando94@gmail.com (N.M.); mpando@fmed.uba.ar (M.A.P.); 2 Departamento de Microbiología, Facultad de Medicina, Universidad de Buenos Aires, Buenos Aires 1121, Argentina; mrosenzvit@fmed.uba.ar; 3 Instituto de Investigaciones en Microbiología y Parasitología Médica (IMPaM, UBA-CONICET), Universidad de Buenos Aires, Buenos Aires 1121, Argentina; 4 Medical Informatics and Bioinformatics, Institute for Measurement Engineering and Sensor Technology, Hochschule Ruhr West, University of Applied Sciences, 45479 Mülheim an der Ruhr, Germany

**Keywords:** MicroRNAs, HIV, HIV miRNAs, MicroRNA regulation, miRNA diagnostics, miRNA therapeutics

## Abstract

MicroRNAs (miRNAs), a class of small, non-coding RNAs, play a pivotal role in regulating gene expression at the post-transcriptional level. These regulatory molecules are integral to many biological processes and have been implicated in the pathogenesis of various diseases, including Human Immunodeficiency Virus (HIV) infection. This review aims to cover the current understanding of the multifaceted roles miRNAs assume in the context of HIV infection and pathogenesis. The discourse is structured around three primary focal points: (i) elucidation of the mechanisms through which miRNAs regulate HIV replication, encompassing both direct targeting of viral transcripts and indirect modulation of host factors critical for viral replication; (ii) examination of the modulation of miRNA expression by HIV, mediated through either viral proteins or the activation of cellular pathways consequent to viral infection; and (iii) assessment of the impact of miRNAs on the immune response and the progression of disease in HIV-infected individuals. Further, this review delves into the potential utility of miRNAs as biomarkers and therapeutic agents in HIV infection, underscoring the challenges and prospects inherent to this line of inquiry. The synthesis of current evidence positions miRNAs as significant modulators of the host-virus interplay, offering promising avenues for enhancing the diagnosis, treatment, and prevention of HIV infection.

## 1. Introduction

Dysregulation of gene expression is a hallmark of disease, and differences in gene expression can be used for diagnosis, prognosis, and monitoring. The modulation of gene expression is a cooperative process among many players, for example, transcription factors, DNA methylation, histone modification, and non-coding RNAs such as microRNAs (miRNAs). The latter are short (18–24) non-coding RNA sequences in their mature form. MicroRNAs are intrinsic molecules transcribed from the genome that act in the post-transcriptional modulation of gene expression [1]. Additionally, miRNAs and similar regulatory RNAs can originate from other sources, such as cell-cell communication, from intracellular pathogens such as Brucella suis [2] and Toxoplasma gondii [3], and also from viruses like Herpesvirus and the human cytomegalovirus [4]. In the following, we will outline the impact of HIV and miRNAs on health. We will then investigate to what extent miRNAs and HIV have been associated and conclude with future perspectives.

## 2. The Impact of HIV on Global Health

Since its recognition in 1981, Human Immunodeficiency Virus/Acquired Immune Deficiency Syndrome (HIV/AIDS) has emerged as a paramount challenge in global health despite HIV screening and antiretroviral treatment [5]. With approximately 40 million individuals affected globally (according to the World Health Organization), the pandemic has exerted a profound influence, particularly in sub-Saharan Africa, accounting for about two-thirds of the worldwide infections. Despite advancements in HIV screening and antiretroviral treatment, sexual transmission prevention remains an impediment.

According to the World Health Organization (WHO), so far, an estimated 40 million people have succumbed to HIV and the AIDS disease it causes. In 2022, more than half a million people died from HIV-related causes, and more than 1 million new infections were estimated [6]. HIV/AIDS, tuberculosis, and malaria are major global health threats, causing substantial morbidity and mortality. Despite increased funding and progress, weak health systems and inadequate resources remain significant obstacles to combating these diseases, highlighting the need for integrated and comprehensive health approaches [7].

HIV/AIDS has rapidly evolved into a global health crisis, with the most significant impact in sub-Saharan Africa. Initiatives such as widespread HIV screening and the provision of antiretroviral treatment have decreased the transmission of HIV. Nevertheless, challenges persist in preventing sexual transmission, underscoring the necessity for innovative approaches [5]. In sub-Saharan Africa, HIV is exacerbated by tuberculosis and malaria, putting large strains on the health systems. The triad of HIV/AIDS, tuberculosis, and malaria leads to high morbidity and mortality rates. Efforts to combat the situation have been made, but more integrated and robust health strategies are needed [7]. A more recent review underlines the need for enhanced methodologies and interventions to this day [8]. Vulnerability to other diseases and their progressions are also impacted by HIV/AIDS, including effects on vaccine efficacy [9]. The infection status of HIV/AIDS patients with *Toxoplasma gondii*, a parasite that, after an acute infection, persists in the host in the form of tissue cysts that can reactivate when the immune system is compromised, shows the broad range of interconnected diseases. HIV/AIDS patients showed an 81% positive status for *T. gondii* IgG, and almost 70% of the studied population had cerebral toxoplasmosis [10]. If left untreated in HIV/AIDS patients, toxoplasmosis is typically fatal [11], showing the grave need for constant screening or medication, given the high infection status among HIV patients. The impact of HIV/AIDS on the mental health of patients is an additional factor that influences outcomes and can further exacerbate immunosuppression, for example, via stress [12].

While detrimental effects on global health and health systems are evident, the secondary effects of HIV/AIDS could be overlooked. A detrimental impact on human development, such as economic progress, educational outcomes, security, and other effects, creates a significant challenge [13,14,15].

Despite considerable research efforts, a fully effective HIV vaccine remains elusive. However, in recent years, understanding the virus’ structure, immune escape mechanisms, and the identification of broadly neutralizing antibodies (bNAbs) have led to novel vaccine designs [16]. Regarding treatments, many antiretroviral therapies are now available, thereby improving life expectancy and quality of life. Recent efforts have focused on long-acting antiretrovirals, which are transformative by reducing the daily pill burden to a few injections per year. Drugs like cabotegravir and rilpivirine represent the forefront of this innovation. They are receiving approval from regulatory bodies in the United States and Canada. Furthermore, islatravir, a nucleoside reverse transcriptase translocation inhibitor, is investigated as a potential implant with a dosing interval of a year or more [17]. In addition to preventive strategies and treatment, detection and disease monitoring are important measures. Point-of-care HIV assays have revolutionized HIV diagnostics, offering rapid onsite detection without the need for a sophisticated laboratory. These advancements are particularly beneficial in resource-limited settings [18]. The detection of HIV is crucial for preventing the spread of the virus. Different methods are needed to monitor the disease, such as the viral load of an already-diagnosed patient. One new method to detect HIV is isothermal nucleic acid amplification, which may also help monitor viral load [19]. The search for novel biomarkers and the application of next-generation sequencing technologies are at the forefront of enhancing HIV diagnostic precision. Biosensors for rapid, portable, and susceptible point-of-care detection of HIV, which provide timely diagnosis and monitoring of infectious agents, are also emerging [20]. Despite advancements in HIV detection and tracking, challenges remain in the widespread adoption of these technologies, especially in resource-limited settings. Future research should focus on developing affordable, robust, easy-to-use, and accurate diagnostic tools. Whether microRNAs (miRNAs) can be used in strategies for diagnosis, monitoring, or even treatment will be discussed later in this work. First, we will introduce this class of molecules in the following section.

## 3. Overview of microRNAs and Their Medical Significance

MicroRNAs are short (18–24 nucleotides) non-coding RNA sequences involved in post-transcriptional modulation of gene expression [21]. They are involved in various biological processes and have been implicated in many diseases [22]. The primary miRNAs (pri-miRNAs) are initially transcribed from their loci or as part of a gene. Thermodynamically, RNA folds upon itself to minimize free energy, and precursor miRNAs (pre-miRNAs), structures that resemble hairpins, are then excised from pri-miRNAs by Drosha in the nucleus. They are channeled to the cytoplasm via Exportin 5, where they are further processed by Dicer and then incorporated in the RNA-induced silencing complex (RISC) as a single-stranded mature miRNA. The mature miRNA acts as a recognition element for its target mRNAs within RISC. The recognition is guided via base pairing between the mature miRNA within RISC and its target mRNA. Upon the binding of RISC, the mRNA is destabilized, or translation efficiency is altered. Also, the mRNA can be sliced, mainly in plants where perfect pairing between the miRNA and its mRNA target site occurs. More information about the genesis and function of miRNAs is available in two books on the topic: miRNomics 2014 [21] and miRNomics 2022 [1]. MicroRNAs are not only present inside the cells. They are also found in bodily fluids and have been found to play a role in cell-cell communication. This easy access to miRNAs in bodily fluids makes it possible that miRNAs can be utilized as biomarkers. On the other hand, their involvement in modulating protein abundance makes them a potential druggable target. In the following, we will discuss the role of miRNAs in communication among cells, their potential as biomarkers, and therapeutics/therapeutic targets.

## 4. Cell-Cell Communication via MicroRNAs

MicroRNAs are involved in virtually all regulatory cellular pathways [23]. In addition, miRNAs are detectable in the blood and other body fluids. Extracellular miRNAs are not only released through apoptosis. They are also actively released, packaged in extracellular vesicles such as exosomes, microvesicles, and associated with RNA-binding proteins [24,25]. Recipient cells can take up secreted miRNAs where they function as endogenous miRNAs, simultaneously regulating multiple target genes, suggesting a novel role for miRNAs in intercellular communication [25,26]. Exosomal miRNAs play a significant role in immune system modulation. Immune cells, such as T-cells, release exosomes containing miRNAs. These packaged miRNAs can influence the function of antigen-presenting cells (APCs), enhancing or suppressing immune responses depending on the context [27]. Cell-cell communication via miRNAs has also been shown to be important in metabolism [28], tissue regeneration [29], and adaptation to physiological stress [30]. Even in breeding, miRNAs have shown potential as predictive tools. In bovine embryonic development, specific miRNAs carried by extracellular vesicles (EVs) secreted by embryos can serve as early biomarkers for developmental competence [31].

It is important to remember that the number of RISC complexes per cell is limited and that miRNAs are likely to compete to be loaded into RISC. Hence, an external signal should introduce many miRNAs to have an impact. We recently listed several open challenges within miRNomics [32], which include the needed quantification of RISC, circulating miRNAs, and many more. MicroRNAs have been quantified within exosomes [33,34], and methods for their quantification have been devised [35,36], but more work is needed to improve our understanding of cell-cell communication via miRNAs. Another issue is that a miRNA can only convey its function in the presence of a limited amount of its target mRNAs. With too many targets or suitable miRNA decoys [37] present, the message would quickly become diluted and perhaps inconsequential [38]. It is essential to consider these caveats when considering cell-cell communication via miRNAs. Such communication can be, as seen above, part of the regulation of homeostasis.

A hallmark of diseases is the dysregulation of cellular genes, and miRNAs may also be dysregulated, leading to further downstream dysregulation of proteins, including transcription factors. For example, the let-7 miRNA family is abundant in extracellular fractions from metastatic gastric cancer cells [39]. Circulating miRNAs may also play roles in other human ailments, such as cardiovascular [40] and autoimmune diseases [41]. MicroRNA dysregulation has been associated with viral infection [42,43] and the cellular response to infection [44]. Since miRNAs are available in bodily fluids, they are promising candidates for biomarkers with little invasiveness.

## 5. MicroRNAs as Biomarkers

Due to miRNA stability in bodily fluids, extracellular miRNAs have emerged as promising non-invasive biomarkers for various diseases. They offer insights into the disease state and progression, facilitating early diagnosis and monitoring of treatment responses [45].

MicroRNAs can act as biomarkers for disease diagnosis and reflect the pathophysiological status of cells in autoimmune conditions, potentially offering targets for therapeutic interventions [41]. MicroRNAs are small molecules available in the cytoplasm, and easily packaged and transported out of the cell. As pointed out above, miRNAs are involved in virtually all molecular pathways; since they can also act as messages in a target cell’s cytoplasm without reaching its nucleus, they are likely to be versatile messenger molecules among cells. In the following, we will provide a few examples without providing extensive details. Detailed accounts are reserved for HIV, which will be discussed in the following sections. Viruses have been shown to encode miRNAs, with which they modulate the host environment in their favor [46,47]. On the other hand, they can take advantage of the host miRNAs. A study on miR-100 revealed significant upregulation of miR-100 expression in hepatitis B-virus (HBV) patients compared to controls. Elevated miR-100 expression was positively correlated with viral load, suggesting that circulating miRNAs like miR-100 could be used as biomarkers for HBV infection and might play a role in the susceptibility and prognosis of HBV infection [42]. Whether these types of regulation are more typical for established human viruses or if they are also features of novel ones such as SARS-CoV-2 [48] remains to be shown. In summary, miRNAs are recognized for their role in modulating immune responses and circulating miRNAs have been proposed as biomarkers for viral infections. They are found in both exosomal and non-exosomal (associated with microparticles or proteins) forms in the blood, suggesting their use in diagnostics and clinical applications across a range of viral infections [49].

Since miRNAs are part of cell-cell communication, they also occur in non-infectious diseases like heart disease. For example, miRNAs can modulate heart disease progression and have emerged as potential biomarkers for such conditions [40]. MicroRNAs are explored as biomarkers for various central nervous system disorders, including neurodegenerative diseases and cancers like gliomas and medulloblastomas, highlighting their role in diagnostics [50]. Especially for various cancers, miRNAs’ diagnostic and prognostic potential continues to be explored. Examples are many and include breast [51,52], pancreatic [53,54], and colorectal cancer [55,56].

MicroRNAs are abundant and easily accessible. They are also often dysregulated in disease and could make promising biomarkers, yet in the past, technical issues with miRNA assays posed challenges [57]. Today, many strategies for miRNA assays have been devised, ranging from specifically adapted reverse transcriptase real-time quantitative polymerase chain reaction approaches [58] to smartphone-based devices for noninvasive detection [59]. Another detection method that can be mentioned among the many that have been developed by now is the thermophoretic detection of exosomal miRNAs, highlighting the feasibility of using these circulating miRNAs as biomarkers for early-stage cancer diagnosis [36].

The above points highlight the feasibility of using exosomal miRNAs as biomarkers. In an extensive panel discussion, we also concluded that miRNAs are promising biomarkers and may provide improved diagnostic and prognostic value [60]. Clinical trials of diagnostic miRNAs further corroborate this conclusion [61].

## 6. MicroRNAs as Drugs or Drug Targets

MicroRNAs are essential regulators of gene expression, with a single miRNA being able to influence entire cellular pathways. Since several miRNAs play critical roles in pathological processes, there is an increasing interest in developing miRNA-based therapeutics. The ability of miRNAs to regulate multiple genes offers an excellent advantage for treating multicausal diseases such as cancer. However, this can also produce unwanted off-target effects, and many strategies have been proposed to avoid this pitfall [62]. MicroRNA-based therapy aims to modulate the level or function of miRNAs involved in pathological processes. MiRNA replacement therapy is a strategy employed to augment the level of miRNA necessary for pathology suppression. It can be achieved by transfection with miRNA mimics, which are double-stranded oligonucleotides that imitate endogenous mature miRNA duplexes [63]. On the other hand, miRNA inhibition aims to down-regulate the level or inhibit the function of miRNAs that are pathological drivers. This regulation can be achieved by incorporating oligonucleotides complementary to the miRNA of interest to block its binding to the mRNA target [64]. Using oligonucleotides as potential drugs is challenging due to possible degradation by intra-or extracellular RNAses, difficulty crossing the cellular membrane, and poor pharmacokinetic and pharmacodynamic properties [65]. Also, these molecules can trigger an innate immune system response. Several chemical modifications and delivery systems are being developed to facilitate the in vivo application of these oligonucleotides [62]. In addition, drug-like small molecules directed at miRNA precursors have been proposed to interfere with mature miRNA biosynthesis [66,67], avoiding the unfavorable properties described. In the case of infectious diseases, the pathogen’s molecules that influence several of its own genes or cellular pathways, such as miRNAs, can be considered drug targets with a greater likelihood of efficacy. For example, miRNAs from helminth parasites were proposed as therapeutic targets [68,69]. The additional advantage of parasitic miRNAs as possible drug targets is that some of them are absent in the mammalian host and thus could be considered selective targets. Molecules complementary to parasitic miRNAs (antimiRs) can be utilized to control parasitic diseases. They could be designed specifically for the target, thereby minimizing off-target effects [69,70,71].

When considering miRNAs as therapeutics, many challenges remain [72], such as the “too many targets for miRNA effect”, which occurs when one miRNA targets tens or even hundreds of genes [73]. While innate miRNAs would typically be expressed at levels that would lead to target competition and diminishing effects with a target number, high doses administered as drugs could lead to unexpected gene expression and untoward side effects. Another challenge concerning the miRNA replacement strategy is the competition and saturation effect between exogenous and endogenous miRNAs for the intracellular machinery, which can also lead to side effects. Despite these challenges, miRNA drugs have exhibited significant efficacy in a range of health conditions, including cancer [74], hepatitis C [75], and heart abnormalities [76]. Currently, only ten miRNA drugs are in clinical trials, with none undergoing phase III, while over 60 siRNA drugs are in complete clinical trial progression, including two approvals [61].

## 7. Role of MicroRNAs in HIV Infection and Pathogenesis

### 7.1. Regulation of HIV Replication by microRNAs

#### 7.1.1. Overview of the HIV-1 Replication Cycle

HIV-1 mainly infects CD4+ T lymphocytes and macrophages. During the acute phase of infection, however, it also infects dendritic cells, and in the chronic phase, it shows a wider tropism, infecting other immune and non-immune cells [77]. The HIV-1 replication cycle can be divided into 11 stages: binding, fusion, reverse transcription, entry into the nucleus, uncoating, integration, transcription, translation, assembly, budding, and release/maturation [78]. The envelope glycoprotein gp120 comes into contact with CD4 expressed on the cell surface, triggering a conformational change that allows it to bind to co-receptors (CCR5 or CXCR4, depending on the tropism of the virus), thus facilitating fusion of the envelope with the cell membrane and release of the viral contents into the cytoplasm. Once in the cytoplasm, partial dissolution of the capsid occurs, which is necessary for the initiation of reverse transcription, i.e., the synthesis of double-stranded viral DNA from the RNA genome. Recent experiments have challenged the common consensus that complete stripping of the viral core is necessary to import the viral genome into the nucleus [79,80]. The viral capsid has been shown to pass intact, or nearly intact through the nuclear pore complex. Thus, reverse transcription is completed in the nucleus under the protection of the capsid, and genome stripping occurs during or after genome release to the integration site. The viral cDNA is integrated into the genome as a provirus, particularly in AT-rich chromatin regions and other transcriptionally active sites, mediated by the viral integrase [78,81,82].

Once integrated into the genome, the virus can follow two pathways: active transcription and production of new virions, or inefficient transcription, resulting in viral latency. In the first case, the virus uses most of the host transcriptional machinery, recruiting specific regulatory proteins to minimize host antiviral activity. In the second case, viral DNA transcription is inhibited, and the cells containing the provirus become a viral reservoir. This integration constitutes the main obstacle to eradicating the infection since the immune system does not efficiently detect the provirus contained in latently infected cells, is unaffected by antiretroviral treatment, and its transcription is reactivated upon cessation of therapy [83,84,85].

A complete messenger RNA (mRNA) containing all the reading frames is generated as a product of provirus transcription. This transcript undergoes different forms of alternative splicing, giving rise to three classes of mature mRNA: (i) complete 9.2 kb transcripts (which do not undergo splicing), which in turn serve as the genome of the new virions and as the mRNA coding for the Gag and Pol polyproteins; (ii) partially spliced transcripts of 4 kb, coding for Env, Vif, and Vpr; and (iii) fully spliced transcripts of 2 kb, coding for Rev, Tat, and Nef. The latter are the predominant species in the early stages. These small mRNAs can be transported to the cytoplasm for translation. The Tat protein enables effective mRNA elongation, while Rev facilitates the export of longer transcripts to the cytoplasm, and Nef modulates intracellular protein trafficking pathways to prevent antigenic presentation as well as facilitate viral particle release and infectivity [86,87,88,89].

Through the activity of Rev, the longer transcripts are translated into the cytoplasm, and the Gag, Pol, Env, Vif, and Vpr proteins are transported to the virion assembly site at the plasma membrane. Upon arrival at the plasma membrane, the Gag protein and the complete genomic RNA are assembled into the nascent, immature viral particle and the GagPol precursor. Gag sequesters the cellular machinery necessary for budding and release of the viral particle, and the virion finally matures when the viral protease cleaves the Gag polyprotein precursor [82].

#### 7.1.2. MicroRNAs Regulate the HIV-1 Replication Cycle

Human miRNAs can regulate the HIV-1 replication cycle by directly targeting, destabilizing, and inhibiting the translation of HIV-1 transcripts or by targeting human transcripts that play a role in the viral replication cycle. In the latter case, the miRNA can contribute to the enhancement or inhibition of viral replication, depending on whether its target functions as an HIV-1 restriction factor or dependency factor [90]. Although individual miRNAs may have pro-viral effects, the finding that Dicer or Drosha silencing/knockdown leads to an increase in HIV-1 production and infectivity suggests that miRNA regulation negatively impacts HIV-1 replication [91,92].

**Figure 1:**
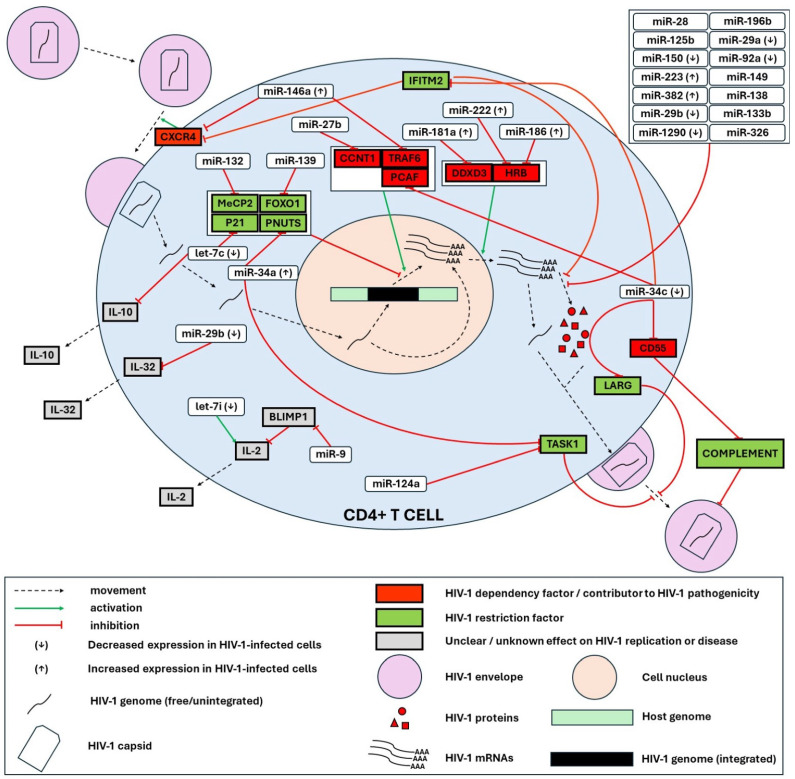
microRNA-mediated HIV-1-human interactions reported in CD4+ T cells and T-cell lines. HIV-1 entry is facilitated by the chemokine receptor CXCR4. CXCR4 is silenced by miR-146a, which is upregulated upon HIV-1 infection, as well as by IFITM2, which is in turn silenced by miR-34c, whose expression is reduced upon HIV-1 infection. The next highly regulated step of HIV-1 replication is the transcription of the integrated provirus. This step is inhibited by the restriction factors MeCP2, FOXO1, PNUTS, and P21, which are silenced by miR-132, miR-139, miR-34a (upregulated upon HIV-1 infection) and let-7c (downregulated upon HIV-1 infection), respectively. HIV-1 transcription is dependent on host factors CCNT1, TRAF6, and PCAF, which are silenced by miR-27b, miR-146a, and miR-34c, respectively. The export of viral mRNA to the cytoplasm is promoted by DDXD3, which is silenced by miR-181 (upregulated upon HIV-1 infection), and by HRB, which is silenced by miR-222 and miR-186 (both upregulated upon HIV-1 infection). The translation of viral mRNA is inhibited by IFITM2, as well as by several miRNAs, some of which are upregulated upon HIV-1 infection (miR-223, miR-382), others downregulated (miR-150, miR-29a, miR-29b, miR-92a, miR-1290) and others with no reported modulation (miR-28, miR-196b, miR-125b, miR-149, miR-138, miR-133b, miR-326). The release and maturation of new HIV-1 particles is inhibited on the one hand by LARG, which is silenced by miR-34c, and on the other hand by TASK1, which is silenced by miR-34a and miR-124a. CD55, which protects the released virion from inactivation by human complement, is silenced by miR-34c. Finally, several cytokines with unclear roles in HIV-1 pathogenesis are regulated by microRNAs: IL-10 by let-7, IL-32 by miR-29b (downregulated upon HIV-1 infection) and IL-2 by let-7i (downregulated upon HIV-1 infection) and by miR-9 (through BLIMP1).

**Figure 2:**
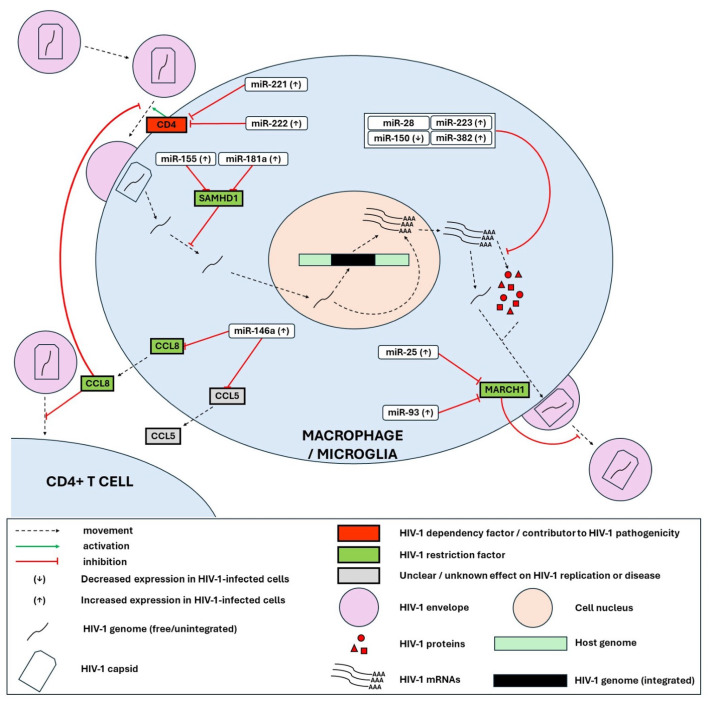
MicroRNA-mediated HIV-1-human interactions reported in macrophages and microglia. HIV-1 entry depends on the CD4 receptor, which is silenced by miR-221 and miR-222 (both upregulated upon HIV-1 infection). CCR5-mediated entry to macrophages/microglia, as well as CD4+ T cells, are inhibited by chemokine CCL8, which is silenced by miR-146a (upregulated upon HIV-1 infection). Reverse transcription of the HIV-1 genome is inhibited by the host restriction factor SAMHD1, which is in turn silenced by miR-155 and miR-181a (both upregulated upon HIV-1 infection). The translation of viral mRNA is inhibited by miR-28, miR-150 (downregulated upon HIV-1 infection), miR-223, and miR-382 (both upregulated upon HIV-1 infection). Packaging of the viral glycoprotein Env into nascent virions is inhibited by MARCH1, which is silenced by miR-25 and miR-93 (both upregulated upon HIV-1 infection). Secretion of chemokine CCL5, which promotes monocyte migration, is inhibited by miR-146a.

**Figure 3:**
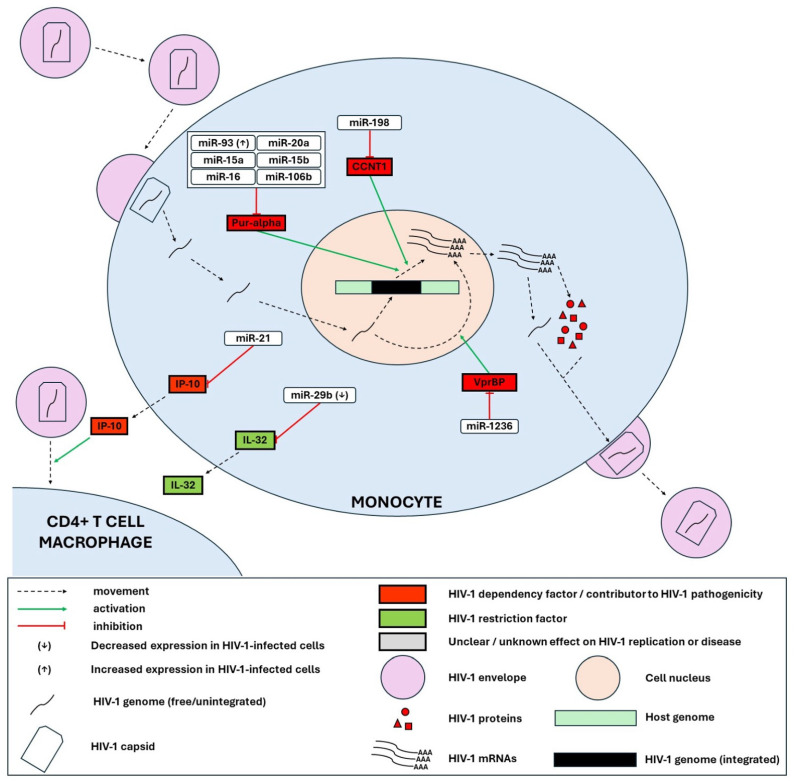
MicroRNA-mediated HIV-1-human interactions reported in monocytes. Monocytes are resistant to HIV-1 infection, as many HIV-1 dependency factors are suppressed by miRNAs. Pur-α, a DNA/RNA-binding protein that promotes HIV-1 transcription in synergy with the viral protein Tat, is maintained at low expression levels through its silencing by miR-15a, miR-15b, miR-16, miR-20a and miR-93 (upregulated upon HIV-1 infection). The HIV-1 dependency factor CCNT1, necessary for viral transcription, is silenced by miR-198. VprBP (or DCAF1) interacts with the viral protein Vpr to direct the degradation of host proteins engaged in epigenetic silencing of the HIV-1 unintegrated genome, thus promoting viral pre-integration transcription. VprBP, which shows low protein-level expression in monocytes compared to macrophages, is silenced by miR-1236, which is more highly expressed in monocytes. The chemokine IP-10 (or CXCL10), which is positively correlated to disease progression and is known to facilitate HIV-1 replication (particularly entry and integration) in CD4+ T cells and macrophages, is silenced by miR-21. IL-32, a pro-inflammatory cytokine known to suppress HIV-1 replication, is silenced by miR-29b, which is upregulated upon HIV-1 infection. miR-125b, miR-28, miR-223, miR-150, and miR-382 have not been conclusively demonstrated to target HIV-1 mRNA directly in monocytes; however, this interaction is likely, given that it has been demonstrated in CD4+ T cells.

In this section, we list all the experimentally validated miRNA-target interactions that may play a role in regulating the HIV-1 replication cycle. We also depict the main miRNA-mediated HIV-1-human interactions reported in CD4+ T cells and T-cell lines (Figure 1), macrophages and microglia (Figure 2) and monocytes (Figure 3). Relevant publications were found by searching the PubMed database with the different combinations of the search terms “microRNA”/“miRNA”/“miR” and “HIV”/“HIV-1”. The miRNA-target interactions were evaluated based on the thoroughness of the experimental validation, considering the following criteria: (i) evidence of co-expression of miRNA and target mRNA in cells, (ii) evidence of interaction between miRNA and the specific miRNA recognition element (mRE) target site, (iii) evidence of miRNA-mediated effects on target protein expression, iv) evidence of miRNA-mediated effects on biological function (i.e., HIV-1 replication) [93]. Table S1 summarizes the miRNAs that have been associated with direct regulation of HIV through post-transcriptional silencing.

Interestingly, a secondary structure seems to block access to the Nef/3′LTR region targeted by miR-223 and miR-29a [94]. Since target site accessibility is essential for miRNA silencing, it has been hypothesized that HIV-1 may have evolved local structures to hamper miRNA targeting [95].

Wang et al. showed that monocytes, which are resistant to HIV-1 infection, express higher levels of mir-28, miR-150, miR-223, and miR-382 compared to macrophages, which are susceptible to infection [96]. These miRNAs had previously been demonstrated by Huang et al. to contribute to HIV-1 latency in resting CD4+ T cells by targeting the HIV-1 3′ LTR [97]. Although these miRNAs were introduced by Wang et al. as 3′ LTR targeting miRNAs, it would be important to test whether they also target the 3′ LTR in monocytes. In a later study [98], Wang et al. showed that morphine, which enhanced HIV-1 replication in monocytes, also down-regulated three of these miRNAs (miR-28, miR-150, and miR-382), as well as miR-125b, which was one of the anti-HIV miRNAs identified by Huang et al., but was not included in the 2009 study by Wang et al. Therefore, while mir-28, miR-150, miR-223, and miR-382 have been shown to be present in resting CD4+ T cells and monocytes, and to prevent productive HIV-1 replication in both cell types, in the case of miR-125b, this has only been proven in resting CD4+ T cells, while in monocytes only its presence has been demonstrated.

Houzet et al. [99] reported that overexpression (transfection) of miR-133b, miR-138, miR-326, miR-149, and miR-92a suppressed HIV-1 replication after 72 h in 42CD4 cells, a cell line derived from 293T, which expresses CD4 and co-receptor molecules. Though the five miRNAs were predicted to have binding sites in the HIV-1 RNA, only the binding of miR-326 to the nef/3′ LTR region was corroborated. The authors did not check for endogenous expression of these miRNAs in 42CD4 or primary T cells since they claim to have selected them among “the top 400 most T-cell-abundant human miRNAs”. Unfortunately, no study of miRNA quantification in T cells is cited as a source. In a more recent study of miRNA expression during human CD4+ T cell early activation [100], miR-133b, miR-326, miR-149, and miR-92a were shown to be expressed in both resting and activated (24 h) CD4+ T cells, whereas miR-138(-5p) was not detected. However, their expression levels are very low (4–3 molecules/cell), compared with more abundant CD4+ T cell miRNAs such as miR-125b-5p (23–29 molecules/cell), let-7a-5p (624–812 molecules/cell) or miR-155-5p (36–1596 molecules/cell). It is therefore uncertain whether the effects of these miRNAs on HIV-1 replication are relevant to the in vivo infection.

All the miRNAs listed in Tables S1 and S2 regulate HIV-1 gene expression by post-transcriptional gene silencing (PTGS). However, Chen et al. [101] reported that miR-146a, which presents no binding sites in HIV-1 transcripts, can bind to Gag protein (specifically, to its RNA-binding domain), preventing Gag multimerization and inhibiting viral particle production. Another example of non-PTGS miRNA regulation is let-7i, which binds to the TATA-box region of the IL-2 promoter, enhancing IL-2 expression and reducing HIV-induced apoptosis in CD4+ T cells [102]. These examples show that miRNAs can regulate HIV-1 replication by other means than PTGS.

Quaranta et al. [103] showed that miR-146a, suppressed by PLZF, targets CXCR4 and TRAF6, inhibiting HIV-1 replication. However, HIV-1 infection did not affect the expression of PLZF, miR-146a, CXCR4, and TRAF6 [103].

Some of the miRNAs included in Table S2 regulate HIV-1 replication or disease progression in an indirect way, through their effect on general immune function, instead of (or in addition to) targeting cellular pathways that affect HIV-1 specifically. One example of this is miR-21 targeting of interferon-γ-induced protein 10 (IP-10 or CXCL-10), a chemokine involved in trafficking immune cells to inflammatory sites, whose plasma levels are up-regulated after HIV-1 infection and positively correlated to disease progression [104]. Among other effects, IP-10 has been shown to enhance latent HIV-1 infection of resting CD4+ T cells and monocyte-derived macrophages in vitro by binding to the chemokine receptor CXCR3 and promoting cofilin activity and actin dynamics, thus facilitating HIV-1 entry and DNA integration [105,106]. However, IP-10 also has more general immunomodulatory effects, e.g., decreasing the production of IFN-γ by PBMC [107], promoting the polarization of naïve CD4+ T cells into the pro-inflammatory Th17 effector profile [108], or affecting T cell retention/migration between lymph nodes and infected sites [109]. Regulation of IP-10 by miR-21 was demonstrated by Wu et al. [110] in monocytes, which are normally resistant to HIV-1 infection. However, since IP-10 is secreted by the monocytes, which are its main producers [104], this constitutes an example of a miRNA regulating HIV-1 replication in one cell type (CD4+ T cells, macrophages) by modulating protein expression in a different cell type (monocytes) through cell-to-cell communication. A similar case is the regulation of CCL5 by miR-146a in macrophages [111]. Suppression of this secreted chemokine by macrophages leads to reduced monocyte migration, which is relevant to HIV-1 infection. However, as the authors clarify, it is unclear whether this would enhance or diminish viral replication or disease progression: on the one hand, recruitment of monocytes is essential for effective control and clearance of infections, while on the other hand, it may also contribute to pathogenic inflammatory processes and increase the available monocytes/macrophage pool for HIV-1 infection [111].

Cyclin T1 constitutes an HIV-1 dependency factor (HDF), since it is the only form of Cyclin T that supports HIV-1 transcription as a subunit of positive transcription elongation factor b (P-TEFb) [112]. Its targeting by miRNAs in monocytes [113] and resting CD4+ T cells [114] partly explains how these cells are refractory to HIV-1 infection. Interestingly, Chiang et al. [114] found that, in resting CD4+ T cells, Cyclin T1 is not only directly regulated by miR-27b, but it is also suppressed by miR-29b, miR-150, and miR-223, which did not show direct targeting of the Cyclin T1 3′ UTR in luciferase reporter assays. The authors hypothesized that miR-150 could be regulating Cyclin T1 through its target c-Myb, but c-Myb knockdown had no effect on Cyclin T1 expression. They attributed the lack of miR-29b effect in the luciferase assay to a potential limitation of their methodology. No comment was made on possible pathways for Cyclin T1 regulation by miR-223. Due to the importance of this protein for HIV-1 replication, it would be interesting to gain insight into how it is regulated by miRNAs.

Sun et al. [94,115] hypothesized that miR-223 and miR-29a/b could regulate HIV-1 replication by targeting cellular targets, in addition to their established direct targeting of HIV-1 transcripts. They validated Sp3, LIF, and RhoB as targets of miR-223 by luciferase reporter assays in HEK293 cells. The authors point to miR-29a/b targets Mcl-1, DNMT3A/B, Tcl1, p85, and CDC42 (which had been experimentally validated previously [116,117,118,119]) as potential pathways for HIV-1 regulation. However, they do not prove co-expression of these miRNAs and their targets in cells susceptible or relevant to HIV-1 infection, do not show modulation of Sp3, LIF, or RhoB protein levels by miR-223 over-expression or silencing, nor do they show that interference with these specific miRNA-target interactions has an effect on HIV-1 replication. These are experiments that should be undertaken in order to verify whether miR-223 and miR-29a/b regulate HIV-1 replication through these cellular mRNAs.

Li et al. [120] identified the chemokine IL-32 as a possible target of the miR-29 family (miR-29a, miR-29b, and miR-29c), but only miR-29b was shown to target IL-32 in luciferase reporter assays. In agreement with this, Monteleone et al. [121] reported that both the miR-29 family and IL-32 are expressed in CD4+ T cells and CD14+ monocytes, but only miR-29b levels are negatively correlated with IL-32 expression in PBMC from treatment-naïve HIV-positive donors. IL-32 is a pro-inflammatory cytokine that induces the antiviral immune response by activating IFN-λ1 and its downstream effectors [120] and has been shown to suppress HIV-1 replication in vitro in PBMC and the pro-monocytic cell line U1 [122,123]. However, in order to verify the miR-29b/IL-32/HIV-1 pathway, in vitro experiments should be carried out over-expressing or suppressing miR-29b in infected CD4+ T cells, macrophages or monocytes while modulating IL-32, to see if there is in fact an IL-32 mediated effect of miR-29b on HIV-1 replication.

Pilakka-Kanthikeel et al. [124] measured miR-181a and miR-155 expression in human astrocytes and found that they were present at similar levels, though low in comparison to microglia. They then showed that inhibition of these miRNAs lead to an increase in the expression of SAMHD1, an HIV-1 restriction factor, while over-expressing them suppresses it. Moreover, they prove that miR-181a and miR-155 inhibition enhances HIV-1 replication in astrocytes. Although they predict miR-181a and miR-155 binding sites in SAMHD1, they do not provide conclusive evidence that their effect on HIV-1 replication in astrocytes is mediated by direct targeting of SAMHD1. Similarly, Shahbaz et al. [125] show that inhibition of miR-30b, miR-30c, and miR-30e in CD8+ T cells causes an increase in CD73 expression, leading to an impaired cytotoxic phenotype and greater migratory capability, but the direct targeting of the CD73 by these miRNAs was not demonstrated through a reporter assay to show direct miRNA-target binding.

Amaral et al. [126] overexpressed miR-34c-5p in Jurkat cells and reported and found six genes downregulated by RT-qPCR: KAT2B/PCAF, JHDM1D, LARG (also known as ARGEF12 or ARHGEF12), CD55, PREX2, and IFITM2. Of these, only KAT2B was validated as a direct target of miR-34c-5p with an effect on HIV-1 replication. It would be interesting to validate whether the remaining genes are direct or indirect targets of miR-34c-5p and whether they affect HIV-1 replication.

Okoye et al. [127] show that plasma EVs from HIV-1-positive donors contain miR-139-5p, that J-Lat cells incubated with these EVs show higher miR-139-5p expression and lower expression of its predicted target FOXO1, as well as reactivation of the latent provirus. However, they do not provide conclusive evidence that miR-139-5p regulates FOXO1 directly or indirectly, or that it is this interaction that leads to viral reactivation.

Swaminathan et al. [128] demonstrated that let-7b, let-7c, and let-7f directly target IL-10 in a T cell line (HUT78), as well as showing that these miRNAs are differentially expressed in CD4+ T cells from healthy donors, chronic HIV-1-infected patients and long-term nonprogressors. However, they fail to see an effect of miRNA overexpression on HIV-1 replication in the HUT78 line, so further research is needed to determine the relevance of this interaction for HIV-1 infection.

Although the scope of this review is limited to miRNAs, it is worth noting that other non-coding RNAs also mediate the host-HIV-1 interaction, sometimes by regulating the miRNA network [129]. For instance, metastasis-associated lung adenocarcinoma transcript 1 (MALAT1), a long non-coding RNA (lncRNA) up-regulated upon HIV-1 infection in macrophages, has been shown to sponge miR-150-5p, leading to an increased expression of SOCS1 (a negative regulator of cytokine signaling), thus promoting HIV-1 replication and reactivation. Silencing of MALAT1 led to the downregulation of miR-155-5p and miR-155-3p [130]. Another lncRNA highly expressed in resting CD4+ T cells, NRON, induces Tat protein degradation, contributing to HIV-1 latency [131]. Zhang et al. [132] reported 18 lncRNA up- or down-regulated upon HIV-1 infection in two T-cell lines. Among these, NEAT1 restricts HIV-1 replication by limiting the cytoplasmic export of HIV-1 mRNAs. Additionally, HIV-1 infection of PBMC and CD4+ T cells induces the expression of the circular RNA (circRNA) ciTRAN, which promotes viral transcription by interacting with the HIV-1 restriction factor SRSF1 [133]. The role of lncRNA, circRNA, and other ncRNA in HIV-1 infection merits further investigation.

### 7.2. Regulation of microRNA Expression by HIV-1

Although it has been proposed that HIV-1 may counter miRNA-mediated restriction by a global suppression of miRNA biogenesis—either by HIV-1 Tat protein inhibition of Dicer or TRBP sequestration by the HIV-1 TAR element [134], other ways of regulation were proposed [135]. For example, it was reported that HIV-1 regulates the expression of individual miRNAs [136]. Bennasser et al. reported that HIV-1 may encode an siRNA [136], but later, Whisnant et al. reported that HIV-1 may neither encode nor interface strongly with cellular miRNAs [137]. Notwithstanding these early reports, research on HIV and miRNAs continues today. The HIV-1 protein Vpr has been shown to interact with Dicer in macrophages, targeting it for proteasomal degradation [138,139]. The possibility of general miRNA suppression by HIV-1 is interesting, considering the findings by Vaidyanathan et al. [140] that cells deficient in miRNA expression or function are rendered more sensitive to death induced by survival gene elimination (DISE) when infected with HIV-1, thus providing a possible additional pathway of HIV-induced apoptosis. General miRNA suppression could also be beneficial to HIV since, as described above, Dicer or Drosha silencing/knockdown led to an increase in HIV-1 production and infectivity which negatively impacts HIV-1 replication [91,92].

A total of 130 instances of in vitro modulation of miRNA expression/secretion by HIV-1 infection or by HIV-1 proteins have been reported, involving 113 different miRNAs (Table S3). The majority were reported in CD4+ T cells (25%), PBMC (24%) and Jurkat cells (15%), while others were reported in macrophages (9%), SupT1 cells (5%), CEM cells (3%), THP-1 cells (3%), HeLa cells (3%), macrophage- and THP-1-derived EVs (3%) and PBMC-derived EVs (11%). In 61% of cases HIV-1 induced miRNA upregulation. miRNA regulation took place mostly in the context of productive HIV-1 infection (69%), latent/reactivated infection (22%) or exposure to HIV-1 proteins (7%), with two instances of modulation in bystander cells (i.e., miR-221 and miR-222 in macrophages [141]).

Boucher et al. [142] showed that HIV-1 infection induces not only miR-155 expression but also the production of miR-155-enriched extracellular vesicles, promoting viral replication in PBMC. Caobi et al. [143] found 17 miRNAs differentially secreted in EVs derived from HIV-1-infected PBMCs, some of which were synergistically modulated by morphine.

Sun et al. [94] hypothesized that suppression of miR-29a and miR-29b in HIV-1-infected cells may be due to Tat-mediated NF-kB activation and Vpr-mediated G2 phase arrest, respectively.

Biswas et al. [144] identified 89 known miRNAs that were significantly down-regulated in PBMC infected with HIV-1 in vitro; of these, we have included in Table S3 only those with at least a 2-fold change in expression.

Several of the studies cited in Tables S1–S3 made use of peripheral blood mononuclear cells (PBMC), either whole or enriched in specific markers [91,92,94,107,142,143,144,145,146]. This is problematic, given that PBMC constitutes a mixed population of several very different cell types: 70–90% lymphocytes (among these 70–85% CD3+ T cells, 5–10% B cells, 5–20% NK cells), 10–20% monocytes, and 1–2% dendritic cells [147]. Thus, the reported effects of miRNAs on HIV-1, or of HIV-1 on miRNAs, emerge from the contribution of all these cell types, making the results harder to interpret. For example, upregulation of miR-4516 in response to productive HIV-1 infection has been independently reported in both PBMC [144] and CD4+ T cells [148] (Table S3); the observation reported in PBMC may be due only to CD4+ T cells, which constitute a major fraction of PBMC. The findings of these studies should ideally be replicated in isolated cell types.

### 7.3. HIV-1-Encoded microRNAs

The presence of miRNAs in the viral genome is a matter of discussion for RNA viruses since canonical processing of the miRNA precursor RNA would result in genome cleavage [149]. Alternative biogenesis pathways could explain the existence of viral miRNAs for such viruses. Several studies have proposed the existence of HIV-encoded miRNAs [90]. For miRNAs derived from the TAR element, miR-TAR, which was reported by several authors [150,151,152,153], a non-canonical biogenesis pathway was proposed. Harwig et al. [154] showed that non-processive transcription from HIV-1 LTR results in the production of ~57 nt hairpin TAR small RNA which can serve as miR-TAR-3p precursor upon DICER cleavage. The main characteristics for a small RNA to be considered a novel miRNA are: (i) length of about 22 nt, (ii) preference for pyrimidine at the 5′ end, (iii) presence of the mature miRNA sequence in a genome sequence that, after transcription, can form a hairpin structure with low free energy with the potential miRNA located in one of the arms of the stem, (iv) presence of RNA sequences differing slightly at the 5′ and 3′ ends, and (v) presence of RNA sequences with partial complementary to the putative miRNA [155]. In the case of miR-TAR-3p, (i) to (iv) were shown by Harwig et al. [154]. In addition, the mature miRNA was shown to be associated with Dicer [151,154] and with Ago, being able to silence RNAs with a complementary target sequence [154]. This viral miRNA was detected in infected cell lines [151,154] and was proposed to contribute to viral latency by recruiting histone deacetylases to the viral LTR promoter [151] while preventing the apoptosis of the host cell by targeting ERCC1 and IER3 [156], as well as Caspase 8, Aiolos, Ikaros and Nucleophosmin (NPM)/B23 [157]. Other HIV miRNAs fulfilling the main characteristic of novel miRNAs were described, for example, miR-N367, named nef-miRNA since it is localized in the nef coding region [158]. However, it is unknown if there exists an alternative biogenesis pathway for these miRNAs. This scenario suggests that more research is needed in order to ascertain whether HIV-encoded miRNAs other than miR-TAR are expressed in infected cells and what would be their role in virus replication and latency.

## 8. MicroRNAs as Biomarkers and Therapeutic Targets in HIV

MicroRNAs have emerged as significant players in post-transcriptional gene regulation. These small, non-coding RNA molecules have been implicated in various biological processes, including viral replication, immune response, and cellular metabolism. Their stability in blood and other bodily fluids makes them promising biomarkers and prognostic markers for HIV infection.

### 8.1. MicroRNAs in HIV Diagnosis

MicroRNAs have shown potential as diagnostic biomarkers for HIV infection. The expression profiles of specific miRNAs change significantly upon HIV infection. Thapa et al. found that altered serum levels of miR-21, miR-122, and miR-223 are seen in HIV-infected individuals, suggesting these miRNAs as biomarkers for HIV infection [159]. Furthermore, Narla et al. demonstrated a unique expression profile of 29 miRNAs in HIV-positive subjects, which could help elucidate viral and immunological control mechanisms and may have diagnostic or prognostic value [160]. Others have also found differentially expressed miRNAs that could be employed as biomarkers: miR-296-5p [161], miR-3162-3p [162], and a small panel consisting of miR-16-5p, miR-20b-5p, miR-195-5p, and miR-223-3p [163] are examples of these efforts.

Another panel of miRNAs (miR-15b-5p, miR-16-5p, miR-20a-5p, miR-26a-5p, miR-126-3p, and miR-150-5p) may help detect patient resistance to highly active antiretroviral therapy [164].

### 8.2. MicroRNAs as Prognostic Markers for HIV

MicroRNAs also hold promise as prognostic markers in HIV infection. Their expression levels correlate with disease progression, immune response, and patient outcomes. Moghoofei et al. assessed the relationship between miRNAs (miR-29, miR-150, miR-155, miR-223) and viral and immunological markers in HIV-1 infection, suggesting their influence on the clinical progression of HIV-1 infection in patients [165]. Swaminathan et al. demonstrated that specific miRNAs are associated with the progression of HIV to acquired immunodeficiency syndrome (AIDS), suggesting their utility in predicting disease progression [166]. Such miRNAs could identify individuals at higher risk of rapid progression, guiding more aggressive treatment approaches or closer monitoring. Yousefpouran et al. (2020) assessed the miRNA profile in viral infections, including HIV mono-infection and co-infections with HBV and HCV. They found differential expression profiles suggesting miRNAs can be biomarkers for disease progression and differentiation among infections [167].

### 8.3. The Potential of microRNAs in HIV Therapy and Prevention Strategies

While there are no direct miRNA-based therapies for HIV, one related area of research (due to high coinfection with HIV) involves the use of miRNA or anti-miRNA strategies in the context of hepatitis C virus (HCV) infection. For instance, a study involving Miravirsen, an anti-miR-122, showed promising results in treating HCV infection by targeting liver-expressed miR-122, essential for HCV replication [168]. This approach of targeting miRNAs to interfere with viral replication could potentially be adapted for HIV treatment, given the regulatory roles miRNAs play in viral infections and host immune responses. MiR-122 levels are also altered significantly during HIV infection [159]. However, as described above, it is necessary to have caution to address the potential side effects of miRNA-based therapeutics, including drugs directed to miR-122 which is further exacerbated by severe side effects of miravirsen which led to the termination of the clinical trial [169].

## 9. Conclusions

In this review, we aimed to provide a detailed and extensive, though not exhaustive, overview of the extant literature on the role played by miRNAs in the context of HIV-1 infection. We placed a special focus on the bidirectional regulation between human miRNAs and HIV-1 and strived to list as many reports as possible of miRNAs found to regulate HIV-1 either by direct targeting of HIV-1 transcripts or by regulating host factors involved in the HIV-1 replication cycle, pathogenesis or immune response, as well as reports of miRNAs whose expression is modulated in the presence of HIV-1 or its proteins. Additionally, we have assessed the thoroughness with which the reported miRNA-target interactions have been experimentally validated to distinguish between high- and low-confidence interactions, thus pointing to concrete experiments that should be carried out to fill the current gaps in knowledge. Finally, for interactions reported long ago, we revised the most recent literature on the target gene function to reassess and update the significance of the interaction.

From the integration of all this data, we make the following observations: (i) overall miRNA regulation by HIV-1 seems to be predominantly positive (see Table S3); (ii) miRNA expression is differentially modulated by HIV-1 in different cell types (e.g., miR-155, miR-148b, miR-221, and miR-21) (see Table S3); (iii) The same miRNA can play pro- or anti-viral roles depending on the cell type (e.g., miR-146a, miR-93) (Figure 1, Figure 2 and Figure 3); (iv) miR-34c seems to be a key player in HIV-1 replication in CD4+ T cells, though with mixed effects, some pro-viral, some anti-viral (Figure 1); (v) HIV-1 transcription and translation seem to be the steps of the replication cycle most controlled by miRNAs (Figure 1, Tables S1 and S2); (vi) Due to cell-to-cell communication via cytokines and EVs, miRNA expression in one cell can impact the replication of HIV-1 in another cell (e.g., miR-21 and IP-10, miR-146a and CCL5) (Figure 2 and Figure 3); (vii) miRNAs can regulate HIV-1 through non-canonical pathways and mechanisms other than PTGS (e.g., upregulation of IL-2 by let-7i and the interaction of miR-146a with gag protein).

As to the state of research on this topic, we observe that (i) a lot of research has been conducted on immune cells, and among these, mainly among CD4+ T cells; more research should be conducted on other immune cells, as well as cells of, e.g., the nervous system; (ii) results reported exclusively in cell lines should be validated in primary cells; (iii) most studies that report miRNA modulation by HIV-1 focus on productive infection, very few in latent infection, and only one in bystander effects; (iv) many studies used PBMC, which should be avoided since it is difficult to draw biological conclusions from such a mixed cell population.

## Supplementary Materials

The following supporting information can be downloaded at: https://www.mdpi.com/article/10.3390/genes15050574/s1, Table S1: microRNAs shown to regulate HIV-1 directly by post-transcriptional gene silencing; Table S2: microRNAs shown to regulate HIV-1 indirectly by post-transcriptional gene silencing; Table S3. microRNAs modulated by HIV-1 infection (productive, latent, or bystander) or HIV-1 proteins. References [84,91,92,94,96,97,98,99,102,103,104,105,106,110,111,113,114,115,116,117,118,120,121,124,125,126,127,128,130,141,142,143,144,145,146,148,170,171,172,173,174,175,176,177,178,179,180,181,182,183,184,185,186,187,188,189,190,191,192,193,194,195] are cited in the supplementary materials.
