# Supplementary material for: The Role of MicroRNAs in HIV Infection
Source: Genes (Basel). 2024 Apr 29;15(5):574. doi: 10.3390/genes15050574 (PMC11120859; doi:10.3390/genes15050574)
Supplement: Supplementary file 1 [file genes-15-00574-s001.zip › genes-2933650-supplementary-done.pdf]

genes

MDPI

Table S1. microRNAs shown to regulate HIV-1 directly by post-transcriptional gene silencing.

miRNA	Target[a]	Cell (state)	Action	Co-expression[b]	Interaction[c]	Effect on target protein expression[d]	Effect on HIV-1 replication[e]	References
miR-28	3′ LTR	CD4+ T cells (resting)	Contributes to HIV-1 latency	✓	✓	✓	✓	[97]
	?	Monocytes	Protects against HIV-1 infection	✓	×	×	✓	[96,98]
miR-29a	Nef/ 3′ LTR	H9 cells, 293T cells, Jurkat cells.	Suppresses viral replication and infectivity	✓	✓	✓	✓	[91,146]
miR-29b	Nef/ 3′ LTR	H9 cells, 293T cells, Jurkat cells.	Suppresses viral replication	✓	✓	✓	✓	[91,99,146]
miR-92a	pol	42CD4 cells	Suppresses viral replication	×	×	✓	✓	[99]
miR-125b	gp160/ gp41/ Rev	CD4+ T cells (resting)	Contributes to HIV-1 latency	✓	✓	✓	✓	[97]
	?	Monocytes	Protects against HIV-1 infection	✓	×	×	×	[98]
miR-133b	gp160/gp41	42CD4 cells	Suppresses viral replication	×	×	✓	✓	[99]
miR-138	gp160/gp41	42CD4 cells	Suppresses viral replication	×	×	✓	✓	[99]

miR-149	gag/p24	42CD4 cells	Suppresses viral replication	×	×	✓	✓	[99]
miR-150	gp160/ gp41/ Rev	CD4+ T cells (resting)	Contributes to HIV-1 latency	✓	✓	✓	✓	[97]
	?	Monocytes	Protects against HIV-1 infection	✓	✓	✓	✓	[96,98]
miR-196b	Nef/ 3′ LTR	Jurkat (productively and latently infected)	Suppresses viral replication and infectivity	✓	✓	✓	✓	[174]
miR-223	Nef/ 3′ LTR	CD4+ T cells (resting)	Contributes to HIV-1 latency	✓	×	×	✓	[97]
	?	Monocytes	Protects against HIV-1 infection	✓	×	×	✓	[96]
miR-326	Nef / 3′ LTR	42CD4 cells	Suppresses viral replication	×	✓	✓	✓	[99]
miR-382	3′ LTR	CD4+ T cells (resting)	Contributes to HIV-1 latency	✓	✓	✓	✓	[97]
	?	Monocytes	Protects against HIV-1 infection	✓	×	×	✓	[96,98]
miR-1290	Site 1: Nef/ 3′ LTR<br>Site 2: 3′ LTR	Jurkat (productively and latently infected)	Suppresses viral replication and infectivity	✓	✓	✓	✓	[174]

a "NA" indicates that the specific target site was not identified.

b "✓" indicates that the co-expression of the microRNA and its proposed target in the cell of interest was demonstrated. "×" indicates that this was not demonstrated.

c "✓" indicates that the specific interaction between the microRNA and its proposed target was demonstrated by a reporter assay. "×" indicates that this was not demonstrated.
d "✓" indicates that microRNA inhibition and/or overexpression was demonstrated to affect target protein expression. "×" indicates that this was not demonstrated.
e "✓" indicates that microRNA inhibition and/or overexpression was demonstrated to affect HIV-1 replication. "×" indicates that this was not demonstrated.

Table S2. microRNAs shown to regulate HIV-1 indirectly by post-transcriptional gene silencing.

miRNA	Target	Cell (state)	Action[a]	Co-expression[b]	Interaction[c]	Effect on target protein expression[d]	Biological effect[e]	References
let-7	IL-10	HUT78 cells	NA	✓	✓	✓	×	[128]
let-7c	P21/CDKN1A	HeLa-CCR5, JLTRG-R5 cells	Enhanced HIV-1 replication	✓	✓	×	✓	[187]
miR-9	BLIMP1	CD4+ T cells	Enhanced IL-2 secretion	✓	✓	✓	✓	[192]
miR-15a	Pur-Alpha	Monocytes	Protects against HIV-1 infection	✓	✓	✓	✓	[172]
miR-15b	Pur-Alpha	Monocytes	Protects against HIV-1 infection	✓	✓	✓	✓	[172]
miR-16	Pur-Alpha	Monocytes	Protects against HIV-1 infection	✓	✓	✓	✓	[172]
miR-17-5p	KAT2B/PCAF	HeLa cells, PBMC	Reduced HIV-1 replication	✓	✓	×	✓	[92]
miR-20a	KAT2B/PCAF	HeLa cells, PBMC	Reduced HIV-1 replication	✓	✓	×	✓	[92]
	Pur-Alpha	Monocytes	Protects against HIV-1 infection	✓	✓	✓	✓	[172]
miR-21	IP-10	Monocytes, THP-1 cells, 293T cells	Excessive inflammation, immune dysfunction, disease progression, reduced HIV-1 replication	✓	✓	✓	✓	[104–106,110]

			in CD4+ T cells and macrophages					
miR-25	MARCH1	Macrophages	Pro-viral (Evasion of HIV-1 restriction by MARCH1)	✓	✓	✓	✓	[178]
miR-27b	Cyclin T1	CD4+ T cells (resting)	Reduced HIV-1 replication	✓	✓	✓	✓	[114]
miR-29a	Mcl-1, DNMT 3A/B, Tcl1, p85, CDC42	Hela, KMCH, A549, HEK293 cells	NA	✓	✓	✓	✓	[94,116–118,177]
miR-29b	Cyclin T1 (indirectly?)	CD4+ T cells (resting)	Reduced HIV-1 replication	✓	✓	×	✓	[114]
	Mcl-1, DNMT 3A/B, Tcl1, p85, CDC42	Hela, KMCH, A549, HEK293 cells	NA	✓	✓	✓	✓	[94,116–118,177]
	IL-32	CD4+ T cells, CD14+ monocytes, HepG2 cells	Suppression of antiviral immune response, enhanced HIV-1 replication	✓	✓	×	×	[120,121]
miR-30b	CD73	CD8 T cells	Impaired cytotoxic phenotype, greater migratory capability	✓	×	✓	✓	[125]
miR-30c	CD73	CD8 T cells	Impaired cytotoxic phenotype, greater migratory capability	✓	×	✓	✓	[125]

miR-30e	CD73	CD8 T cells	Impaired cytotoxic phenotype, greater migratory capability	✓	×	✓	✓	[125]
miR-34a	SIRT1	TZM-bl	Increased Tat-induced LTR transactivation	✓	✓	✓	✓	[185]
	PNUTS	HEK293T, TZM-bl, MOLT-3, Jurkat cells	Enhanced HIV-1 replication	✓	✓	✓	✓	[186]
	TASK1	HeLa-CCR5, JLTRG-R5 cells	Enhanced HIV-1 replication	✓	✓	✓	✓	[187]
miR-34c-5p	KAT2B/PCAF	CD4+ T cells (naive TCR-stimulated)	Enhanced HIV-1 replication	✓	✓	✓	✓	[126]
	JHDM1D, ARGEF12, CD55, PREX2, IFITM2	Jurkat cells	NA	×	×	✓	×	[126]
miR-93	MARCH1	Macrophages	Enhanced HIV-1 replication (Evasion of HIV-1 restriction by MARCH1)	✓	✓	✓	✓	[178]
	Pur-Alpha	Monocytes	Protects against HIV-1 infection	✓	✓	✓	✓	[172]
miR-106b	Pur-Alpha	Monocytes	Protects against HIV-1 infection	✓	✓	✓	✓	[172]
miR-124a	TASK1	HeLa-CCR5, JLTRG-R5 cells	Enhanced HIV-1 replication	✓	✓	✓	✓	[187]

miR-132	MeCP2	Jurkat cells	Enhanced HIV-1 replication	✓	✓	×	✓	[189,190]
miR-139-5p	FOXO1	J-Lat cells	Reactivation of latent provirus	✓	×	×	×	[127]
miR-146a	CXCR4	CD4+ T cells (resting, activated)	Reduced HIV-1 replication	✓	✓	✓	✓	[103,173]
	TRAF6	CD4+ T cells (resting, activated)	Reduced HIV-1 replication	✓	✓	✓	✓	[103,179,180]
	CCL5	Macrophages	Reduced monocyte migration	✓	✓	✓	✓	[111]
	CCL8/MCP-2	Microglia	Enhanced HIV-1 replication	✓	✓	✓	✓	[181]
miR-150	Cyclin T1 (indirectly)	CD4+ T cells (resting)	Reduced HIV-1 replication	✓	×	✓	✓	[114]
miR-155	TRIM32, LEDGF, ADAM10, TNPO3, Nup153,INPP5D, SOCS1, SAMHD1, PU.1, many others	MDM	Reduced HIV-1 replication	✓	✓	✓	✓	[84,124,170,171,175,176]
	SAMHD1	Astrocytes, microglia	Enhanced HIV-1 replication	✓	×	✓	✓	[124]
miR-181-5p	DDX3X	PBMC, Jurkat, H9-IIIB cells	Reduced HIV-1 replication	✓	✓	✓	✓	[145]

	SAMHD1	Astrocytes, microglia	Enhanced HIV-1 replication	✓	×	✓	✓	[124]
miR-182	NAMPT	TZM-bl cells	Increased Tat-induced LTR transactivation	✓	✓	✓	✓	[191]
miR-186	HRB, HIVEP2	Sup-T1, Jurkat cells, HeLa MAGI-CCR5 cells	Reduced HIV-1 replication	✓	✓	✓	✓	[182]
miR-198	Cyclin T1	Monocytes	Reduced HIV-1 replication	✓	✓	✓	✓	[113]
miR-210	Dicer1, HIVEP2	Sup-T1,Jurkat cells, HeLa MAGI-CCR5 cells	Reduced HIV-1 replication	✓	✓	✓	✓	[182]
miR-217	SIRT1	MAGI cells	Increased Tat-induced LTR transactivation	✓	✓	✓	✓	[184]
miR-221	CD4	Macrophages	Inhibited HIV-1 entry	✓	✓	✓	✓	[141]
miR-222	HRB, Dicer1	Sup-T1,Jurkat cells, HeLa MAGI-CCR5 cells	Reduced HIV-1 replication	✓	✓	✓	✓	[182]
	CD4	Macrophages	Inhibited HIV-1 entry	✓	✓	✓	✓	[141]
miR-223	Cyclin T1 (indirectly)	CD4+ T cells (resting)	Reduced HIV-1 replication	✓	×	✓	✓	[114]
	Sp3, LIF, RhoB	HEK293 cells	?	×	✓	×	×	[94,115]

miR-1236	VprBP	Monocytes	Protects against HIV-1 infection	✓	✓	✓	✓	[188]

a "NA" indicates that the effect on HIV-1 replication or disease was not described.

b "√" indicates that the co-expression of the microRNA and its proposed target in the cell of interest was demonstrated. "×" indicates that this was not demonstrated.

c "√" indicates that the specific interaction between the microRNA and its proposed target was demonstrated by a reporter assay. "×" indicates that this was not demonstrated.

d "√" indicates that microRNA inhibition and/or overexpression was demonstrated to affect target protein expression. "×" indicates that this was not demonstrated.

e "√" indicates that microRNA inhibition and/or overexpression was demonstrated to affect HIV-1 replication or biological function relevant to HIV-1 infection. "×" indicates that this was not demonstrated.

Table S3. microRNAs modulated by HIV-1 infection (productive, latent, or bystander) or HIV-1 proteins.

**UP-REGULATED BY HIV-1**			
**miRNA**	**Cell (state)**	**Regulation**	**References**
miR-9	SupT1 cells	productive infection	[182]
miR-10a-5p	CD4+ T cells	latent or reactivated infection	[194]
miR-15a-5p	PBMC-derived EVs	productive infection	[143]
miR-15b-5p	PBMC-derived EVs	productive infection	[143]
miR-16-2-3p	CD4+ T cells	latent or reactivated infection	[194]
miR-21	Macrophages, THP-1 cells	productive infection, Tat & gp120	[193]
	Macrophage- and THP-1 cell-derived EVs	Tat & gp120	[193]
miR-23a	Macrophages, THP-1 cells	productive infection, Tat & gp120	[193]
	Macrophage- and THP-1 cell-derived EVs	Tat & gp120	[193]
miR-25	Macrophages	Vpu	[178]
miR-27a	Macrophages, THP-1 cells	productive infection, Tat & gp120	[193]
	Macrophage- and THP-1 cell-derived EVs	Tat & gp120	[193]
miR-33b-5p	Macrophages, others	productive infection	[183]
miR-34a	Jurkat cells	productive infection	[92]
miR-93	Macrophages	Vpu	[178]

miR-96-5p	CD4+ T cells	latent or reactivated infection	[194]
miR-98	CD4+ T cells	latent infection	[148]
miR-99b-5p	CD4+ T cells	latent or reactivated infection	[194]
miR-106a-5p	CD4+ T cells	latent or reactivated infection	[194]
miR-107	CD4+ T cells	latent or reactivated infection	[194]
miR-122a	Jurkat cells	productive infection	[92]
miR-125a-5p	CD4+ T cells	latent or reactivated infection	[194]
miR-142-5p	CD4+ T cells	latent or reactivated infection	[194]
miR-142-3p	PBMC-derived EVs	productive infection	[143]
miR-146a	Macrophages	productive infection	[111]
miR-148a-5p	CD4+ T cells	latent or reactivated infection	[194]
miR-148a-3p	CD4+ T cells	latent or reactivated infection	[194]
miR-148b	Macrophages	productive infection	[144]
miR-150-3p	CD4+ T cells	latent or reactivated infection	[194]
miR-151a-5p	CD4+ T cells	latent or reactivated infection	[194]
miR-155-5p	PBMC	productive infection	[142]
	Macrophages, THP-1 cells	productive infection, Tat & gp120	[130,193]
	Macrophage- and THP-1 cell-derived EVs	Tat & gp120	[193]
miR-155-3p	Macrophages	productive infection	[130]

miR-181a-5p	PBMC-derived EVs	productive infection	[143]
miR-185a-5p	CD4+ T cells	latent or reactivated infection	[194]
miR-186	SupT1 cells	productive infection	[182]
miR-192-5p	CD4+ T cells	latent or reactivated infection	[194]
miR-192	SupT1 cells	productive infection	[182]
miR-200c	SupT1 cells	productive infection	[182]
miR-206	Jurkat cells	productive infection	[92]
miR-210-5p	CD4+ T cells	latent or reactivated infection	[194]
miR-210	Jurkat cells	productive infection	[92]
miR-210	SupT1 cells	productive infection	[182]
miR-221	Macrophages	bystander	[141]
miR-222	SupT1 cells	productive infection	[182]
	Macrophages	bystander	[141]
miR-223	CD4+CD8- PBMC, CEM cells, Jurkat cells	productive infection	[94]
miR-297	Jurkat cells	productive infection	[92]
miR-320a	CD4+ T cells	latent or reactivated infection	[194]
miR-320	Jurkat cells	productive infection	[92]
miR-342-3p	CD4+ T cells	latent or reactivated infection	[194]
miR-361-5p	SupT1 cells	productive infection	[182]

miR-370	Jurkat cells	productive infection	[92]
miR-373-3p	Jurkat cells	productive infection	[92]
miR-382	Jurkat cells	productive infection	[92]
miR-383	Jurkat cells	productive infection	[92]
miR-424-3p	CD4+ T cells	latent or reactivated infection	[194]
miR-485	Jurkat cells	productive infection	[92]
miR-497-5p	CD4+ T cells	latent or reactivated infection	[194]
miR-590-3p	CD4+ T cells	latent or reactivated infection	[194]
miR-1246	PBMC-derived EVs	productive infection	[143]
miR-1268a	CD4+ T cells (naive TCR-stimulated)	productive infection	[126]
miR-1273g-3p	CD4+ T cells	latent or reactivated infection	[194]
miR-1275	CD4+ T cells	latent or reactivated infection	[194]
miR-1307-5p	CD4+ T cells	latent or reactivated infection	[194]
miR-3195	PBMC	productive infection	[144]
miR-3613-5p	CD4+ T cells	latent or reactivated infection	[194]
miR-3653-3p	PBMC	productive infection	[144]
miR-3656	PBMC	productive infection	[144]
miR-4488	CD4+ T cells	latent infection	[148]

	PBMC	productive infection	[144]
miR-4492	PBMC	productive infection	[144]
miR-4516	CD4+ T cells	latent infection	[148]
	PBMC	productive infection	[144]
miR-4677-3p	CD4+ T cells	latent or reactivated infection	[194]
miR-4697-3p	CD4+ T cells	latent or reactivated infection	[194]
miR-6087	PBMC	productive infection	[144]
miR-7974	CD4+ T cells	latent infection	[148]
	**DOWN-REGULATED BY HIV-1**		
**miRNA**	**Cell (state)**	**Regulation**	**References**
let-7d	PBMC	productive infection	[144]
let-7i	CD4 T cells	productive infection	[102]
miR-7-1	PBMC	productive infection	[144]
miR-7-2	PBMC	productive infection	[144]
miR-7-3	PBMC	productive infection	[144]
miR-16	HeLa cells	productive infection	[195]
miR-17/92 cluster	Jurkat cells	productive infection	[92]

(miR-17-5p, miR-17-3p, miR-18, miR-19a, miR-20a, miR-19b-1, miR-92-1)			
miR-21	CD4+CD8- PBMC	productive infection	[94]
miR-26a-2-3p	PBMC	productive infection	[144]
miR-29a	CD4+CD8- PBMC, CEM cells, Jurkat cells	productive infection	[94]
miR-29b	CD4+CD8- PBMC, CEM cells, Jurkat cells	productive infection	[94]
miR-29c	CD4+CD8- PBMC, CEM cells, Jurkat cells	productive infection	[94]
miR-34c-5p	CD4+ T cells (naive TCR-stimulated)	productive infection	[126]
miR-92b-3p	PBMC	productive infection	[144]
miR-93	HeLa cells	productive infection	[195]
miR-126-5p	CD4+ T cells (naive TCR-stimulated)	productive infection	[126]
miR-126-3p	CD4+ T cells (naive TCR-stimulated)	productive infection	[126]
miR-143-3p	CD4+ T cells (naive TCR-stimulated)	productive infection	[126]
miR-148b	HeLa cells	productive infection	[195]
miR-150-5p	Macrophages	productive infection	[130]
miR-155-5p	CD4+ CD8- PBMC, CEM cells, Jurkat cells	productive infection	[94]

miR-185-5p	PBMC	productive infection	[144]
miR-191-5p	PBMC	productive infection	[144]
miR-199a-1	PBMC	productive infection	[144]
miR-199a-2	PBMC	productive infection	[144]
miR-221	HeLa cells	productive infection	[195]
miR-371a-5p	PBMC-derived EVs	productive infection	[143]
miR-379-5p	CD4+ T cells<br>(naive TCR-stimulated)	productive infection	[126]
miR-411-5p	PBMC-derived EVs	productive infection	[143]
miR-484	PBMC	productive infection	[144]
miR-502-5p	PBMC-derived EVs	productive infection	[143]
miR-548aa +miR-548t-3p	PBMC-derived EVs	productive infection	[143]
miR-570-3p	PBMC-derived EVs	productive infection	[143]
miR-603	PBMC-derived EVs	productive infection	[143]
miR-627-5p	PBMC-derived EVs	productive infection	[143]
miR-644a	PBMC-derived EVs	productive infection	[143]
miR-671-3p	PBMC	productive infection	[144]
miR-671-5p	PBMC	productive infection	[144]
miR-1253	PBMC-derived EVs	productive infection	[143]

miR-1273h-5p	PBMC	productive infection	[144]
miR-1273h-3p	PBMC	productive infection	[144]
miR-1290	PBMC-derived EVs	productive infection	[143]
miR-1301-3p	PBMC	productive infection	[144]
miR-4531	PBMC-derived EVs	productive infection	[143]
miR-4536- 5p	PBMC-derived EVs	productive infection	[143]
